# Urinary Levels of IL-1*β* and GDNF in Preterm Neonates as Potential Biomarkers of Motor Development: A Prospective Study

**DOI:** 10.1155/2017/8201423

**Published:** 2017-05-02

**Authors:** Rafael Coelho Magalhães, Janaina Matos Moreira, Érica Leandro Marciano Vieira, Natália Pessoa Rocha, Débora Marques Miranda, Ana Cristina Simões e Silva

**Affiliations:** ^1^Laboratório Interdisciplinar de Investigação Médica, Faculdade de Medicina, Universidade Federal de Minas Gerais (UFMG), Belo Horizonte, MG, Brazil; ^2^Instituto Nacional de Ciência e Tecnologia de Medicina Molecular, Faculdade de Medicina, UFMG, Belo Horizonte, MG, Brazil

## Abstract

*Objectives*. To evaluate the association between inflammatory biomarkers, neurotrophic factors, birth conditions, and the presence of motor development abnormalities in preterm neonates. *Methods*. Plasma and urinary levels of cytokines (IL-1*β*, IL-6, IL-10, TNF, and IL-12p70), chemokines (CXCL8/IL-8, CCL2/MCP-1, CCL5/RANTES, CXCL10/IP-10, and CXCL9/MIG), and neurotrophic factors (BDNF and GDNF) were evaluated in 40 preterm neonates born between 28 and 32 incomplete weeks of gestation, at four distinct time points: at birth (umbilical cord blood) (T0), at 48 (T1), at 72 hours (T2), and at 3 weeks after birth (T3). Biomarkers levels were compared between different time points and then associated with Test of Infant Motor Performance (TIMP) percentiles. *Results*. Maternal age, plasma, and urinary concentrations of inflammatory molecules and neurotrophic factors were significantly different between groups with normal versus lower than expected motor development. Higher levels of GDNF were found in the group with lower than expected motor development, while IL-1*β* and CXCL8/IL-8 values were higher in the group with typical motor development. *Conclusion*. Measurements of cytokines and neurotrophic factors in spot urine may be useful in the follow-up of motor development in preterm neonates.

## 1. Introduction

Preterm labor and related conditions are associated with systemic inflammatory process in the fetus or neonate [1–6], which, in turn, may contribute to early brain injury [6, 7]. Brain injury is a frequent perinatal complication and an important risk factor for long-term abnormalities of neurodevelopment [8]. Fetal and neonatal nervous systems are highly sensible to inflammation [2, 8–11]. Increased inflammatory response supposedly results in cell death within the nervous tissue in preterm neonates, as observed in experimental models [4, 5, 12, 13] and in post mortem findings [14]. These events may translate into the occurrence of clinical conditions including seizures, sensory disturbances, cognitive limitations, and cerebral palsy [2, 15–19]. Premature neonates often have altered motor skills, ranging from abnormalities of posture and movement to restrictions in fine motor skills [20–22]. Inflammation-related neuronal lesion may culminate in early brain injury [7, 10, 23]. Most cellular interactions in this process, including microglial activation [4, 5, 14], are mediated by cytokines and chemokines released under the influence of different stimuli [24, 25]. One possible mechanism would be the induction of endothelial damage in the white matter, favoring increased neuronal and glial cell apoptosis [25]. There is also interference with the axons' growth, the formation of the myelin sheath [4, 10], and disturbances in neuronal migration, division, organization, and development of synapses [25].

Brain development following an injury is neither static nor a direct consequence of a single event; it actually associates to innumerous cellular and molecular cascades [26]. The resulting balance between injury-associated inflammation and protective mechanisms, including the production of neurotrophic factors [27], is critical for the final development of the central nervous system (CNS) [28]. In this context, we hypothesize that inflammation is associated with motor development in preterm infants. In order to evaluate the association between inflammatory molecules, clinical characteristics, and motor development abnormalities, plasma and urinary levels of cytokines, chemokines, and neurotrophic factors were evaluated in preterm neonates of 28 to 32 incomplete weeks of gestational age at different time points. These measurements were associated with motor development assessed by the Test of Infant Motor Performance (TIMP).

## 2. Patients and Methods

### 2.1. Study Design and Subjects

This was a prospective observational study of preterm neonates (PTN) from 28 to 32 incomplete weeks of gestational age, who were born from June to December 2014 in a philanthropic hospital in Minas Gerais/Brazil. Infants who were admitted to the neonatal intensive care unit (NICU) and whose parents signed the free and informed consent were enrolled.

Exclusion criteria were (i) 5‐minute Apgar score below 7; (ii) diagnosis of congenital malformations, syndromes, and/or associated diseases; (iii) presence of an acute disorder, including sepsis or necrotizing enterocolitis, at any of the time points; and (iv) death within the first three weeks of life.

Gestational age and birth weight, gender, Apgar scores, infant's diagnosis at admission in the NICU, conditions associated with the premature birth, and antenatal exposure to glucocorticoids were collected from hospital data.

The study was approved by the Ethics Committee of both the Federal University of Minas Gerais and the Sofia Feldman Hospital. The study protocol did not interfere with medical recommendations or the treatment of preterm neonates in the NICU.

### 2.2. Study Protocol

All study participants had biological samples collected at the following time points: at birth (cord blood), 48 and 72 hours of life, and at 3 weeks after birth. Motor development of the neonates was evaluated by means of the Test of Infant Motor Performance (TIMP) that was performed when the babies reached 34 weeks of gestational age.

### 2.3. Blood Samples

Umbilical cord blood (5 mL) was collected in sodium heparin tubes (T0). All other samples were obtained simultaneously with other routine laboratory tests in the NICU, without the need for extravenous punctures. At 48 hours (T1), 72 hours (T2), and 3 weeks after birth (T3), venous blood (1 mL) was collected in tubes containing sodium heparin. All samples were immediately centrifuged (5000 rpm, 10 minutes, room temperature), and plasma aliquots were stored at −80°C until assayed.

### 2.4. Urine Samples

Urine samples were obtained at the same time points of peripheral blood collection after birth by using a newborn urinary collector, at 48 and 72 hours (T1 and T2, respectively) and at 3 weeks after birth (T3). Urine samples were transferred to 15 mL plastic tubes and immediately centrifuged (3800 rpm, 5 minutes, room temperature). The supernatant was collected and stored at −80°C until analysis.

### 2.5. Measurement of Plasma and Urinary Concentrations of Chemokines, Cytokines, and Neurotrophic Factors

Measurements of cytokines and chemokines in plasma and urine were performed by cytometric bead array (CBA), according to the manufacturer's instructions (BD Biosciences, San Diego, CA, USA), using kits for quantitation of inflammatory proteins [*CBA Human Inflammatory Kit*: interleukin‐ (IL‐) 1*β*, IL‐6, IL‐10, tumor necrosis factor (TNF), and IL‐12p70] and chemokines [*CBA Human Chemokine Kit*: CXCL8/IL‐8, CCL2/monocyte chemoattractant protein‐1 (MCP‐1), CCL5/regulated on activation, normal T expressed and secreted (RANTES), CXCL10/interferon‐*γ*‐induced protein‐10 (IP‐10), CXCL9/monokine‐induced by interferon‐*γ* (MIG)]. Briefly, plasma and urine samples were incubated in capture microspheres coated with antibodies that were specific for the respective cytokines and chemokines as well the standard curve proteins. The color reagent (phycoerythrin (PE)) was then added, and the samples were incubated at room temperature for 3 hours. After incubation, samples were washed (Wash buffer®, BD Biosciences) and centrifuged (200*g*, 5 min, room temperature). The supernatant was discarded, and the pellet containing the beads was again suspended in the wash buffer (300 *μ*L). Acquisition was performed in a FACSCanto II flow cytometer (BD Biosciences). The instrument has been checked for sensitivity and overall performance with Cytometer Setup & Tracking beads (BD Biosciences) prior to data acquisition. Quantitative results were generated using FCAP Array v1.0.1 software (Soft Flow Inc., Pecs, Hungary). All results are expressed as pg/mL.

Neurotrophic factors [brain‐derived neurotrophic factor (BDNF) and glial cell‐derived neurotrophic factor (GDNF)] levels were determined by enzyme immunoassay (ELISA), following the manufacturer's recommendations (R&D Systems, MN, USA). Briefly, monoclonal antibodies, specific for each neurotrophic factor, were incubated in 96‐well plates for 12–18 hours at 4°C. The plates were then washed 3 times with 300 *μ*L of wash buffer (phosphate buffered saline (PBS) solution, pH 7.4) containing 0.05% of Tween 20). Nonspecific binding sites were blocked with 200 *μ*L per well of PBS solution containing 1% bovine serum albumin (BSA) and incubated for 2 hours at room temperature. Plates were washed with wash buffer, and samples or standards were added to the plates. After incubating for 18 hours at 4°C, plates were then washed and the detection antibody was added to each well, remaining for 2 hours. Plates were washed again, and streptavidin‐peroxidase solution was incorporated, with subsequent incubation for 30 minutes at room temperature. Plates were washed once more, and the chromogen substrate [o‐phenylendiamine (OPD), Sigma‐Aldrich, St. Louis, MO, USA] was added, diluted in citrate buffer (pH 5.0) containing 0.02% H2O2 30 volumes (Sigma‐Aldrich). Finally, plates were incubated in the dark for 30 minutes at room temperature. Reaction was stopped with 1M sulfuric acid solution. Plates were read at 492 nm in a spectrophotometer (Emax, Molecular Devices).

All samples were assayed in duplicate in a single assay to avoid interassay variation. The CBA kits used for the simultaneous quantification of cytokines and chemokines have intra‐assay variations between 4 and 13% for IL‐6, IL‐1*β*, IL‐8, IL‐10, and TNF‐*α* and between 3.4 and 13.9% for CXCL8/IL‐8, CCL2/MCP‐1, CCL5/RANTES, CXCL10/IP‐10, and CXCL9/MIG, respectively. Our intra‐assay variation in CBA experiments ranged from 3 to 6% for all measurements. Neurotrophic factors were measured by ELISA with an intra‐assay variation of about 5% for all measurements.

### 2.6. Motor Development Evaluation

The Test of Infant Motor Performance (TIMP) is a functional motor behavior test used in infants that analyzes child posture and motion. TIMP can be used once the newborn reaches 32 weeks of gestational age and up to four months of corrected age [29]. In addition, TIMP is sensitive to changes in motor coordination, according to age, and discriminates among infants with comorbidities, like brain insults, who generally have lower scores than healthy children [30–32]. TIMP evaluates selective and postural controls, which are needed for functional movement in children, including movements used for exploration and interaction with the environment. The scale is divided into 13 observed items (present or absent response) and 29 elicited items (rated on a scale ranging from 4 to 7 levels). TIMP items were found to be highly accurate to evaluate motor development and, therefore, are considered reliable in discriminating children who are at risk for different kind of motor impairment [30–32].

In the current study, TIMP was applied when participants reached at least 34 weeks of postmenstrual age. The raw score consisted of the sum of the points obtained in each scale item. Raw scores were converted into *Z*‐scores and percentiles, and the TIMP guidelines for TIMP interpretation were established as “developmental delay” *Z*‐score values below −0.5 and below the 5th percentile. Infants' development was consequently classified in two groups: “typical motor development” (values equal or above the 5th percentile) and “lower than expected” (values below the 5th percentile).

### 2.7. Statistical Analysis

Statistical analysis was performed by the statistical software SPSS version 20.0 (IBM, 2012) and Medcalc version 12.2.1.0 (MedCalc Software, 2012). Continuous variables were described using measures of central tendency and dispersion, and qualitative variables were expressed as absolute frequencies and percentages. Normality was verified using the Shapiro Wilk test. For the time point analysis, the Friedman test was chosen, and for variables with values *p* < 0.05, the Wilcoxon test with Bonferroni correction was utilized.

Correlation analysis between plasma and urine values, intra‐class correlation coefficient (ICC), Spearman correlation, and Bland Altman plot were used, and receiver operating characteristic (ROC) curves for the two samples were adjusted, in order to choose between the plasma and urine markers to be used in association with the motor development.

Groups with different motor development were compared with *t*-test, Mann–Whitney test, chi‐square test of Pearson, and asymptotic chi‐square test of Pearson exact, according to parametric or nonparametric distribution. The Spearman correlation coefficient was used for correlation analysis. Model fit in the logistic regression was evaluated with the Hosmer and Lemeshow test.

To verify possible confounding factors, the analyses were done with all participants and with the exclusion of three neonates who were not previously exposed to corticosteroids.

## 3. Results

### 3.1. Clinical Characteristics

This study enrolled 40 infants, 18 (45%) females and 22 (55%) males. Participants were born from 28 to 32 incomplete weeks of gestational age. Prenatal and birth conditions are shown in Table 1. Mean birth weight was 1477 grams; 10 infants had very low birth weight, and 4 had extremely low birth weight. Respiratory distress syndrome occurred in 60% of the infants, requiring respiratory support as supplemental oxygen and/or continuous positive airway pressure (CPAP). All deliveries were by cesarean section. The most frequent maternal condition associated with premature birth was preeclampsia (80%), followed by other causes including premature placental abruption and rupture of the amniotic sac (20%). Half of the mothers received magnesium sulfate and corticosteroids 24 hours before delivery, 42.5% received only corticosteroids 24 hours before delivery, and 7.5% did not receive any medication.

### 3.2. Plasma and Urinary Concentrations of Inflammatory Biomarkers and Neurotrophic Factors

**Figure 1:**
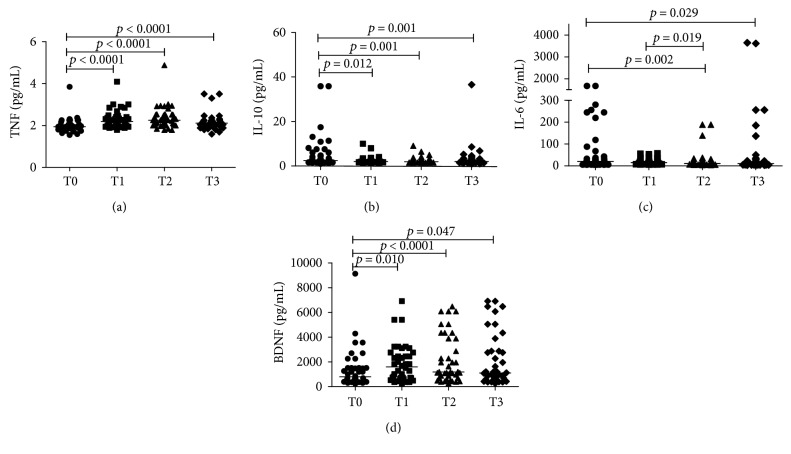
Plasma levels of TNF (a), IL-6 (b), IL-10 (c), and BDNF (d), in preterm infants (*N* = 40) at four different time points (T0: umbilical cord blood, T1: 48 hours, T2: 72 hours, and T3: 3 weeks after birth). CBA was used in ex vivo analysis for cytokines and ELISA for BDNF. Mann–Whitney test was used for comparisons between medians of patients with normal motor development versus patients of lower than expected motor development.

**Figure 2:**
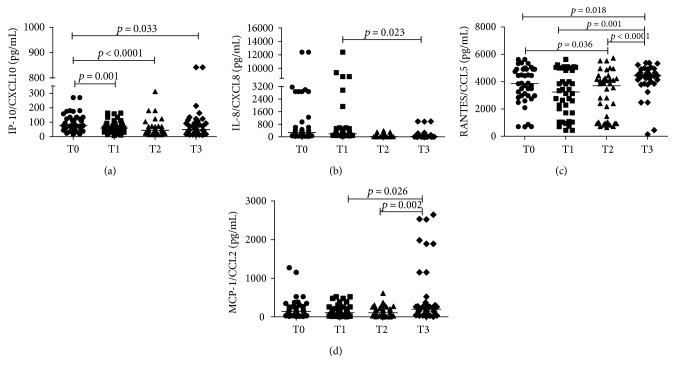
Plasma levels of CXCL8/IL-8, CXCL10/IP-10, CCL5/RANTES, and CCL2/MCP-1 in preterm infants (*N* = 40) at four different time points (T0: umbilical cord blood, T1 48 hours, T2: 72 hours, and T3: 3 weeks after birth). CBA was used in ex vivo analysis. Mann–Whitney test was used for comparisons between medians of patients with normal motor development versus patients of lower than expected motor development.

Newborns presented a significantly decrease in plasma levels of IL‐6, IL‐10, CXCL8/IL‐8, and CXCL10/IP‐10 during the first three weeks of life. The opposite occurred with TNF, CCL2/MCP‐1, CCL5/RANTES, and BDNF. There was a more pronounced increase of TNF and BDNF 48 h after birth (T1). Regarding IL‐12p70, IL‐1*β*, CXCL9/MIG, and GDNF, no differences were observed between different time points. Significant results are shown in Figures 1 and 2.

**Figure 3:**
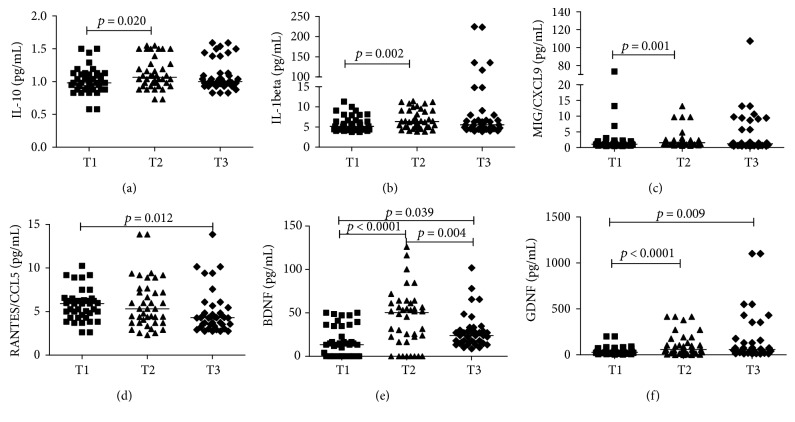
Urinary levels of IL-10, IL1-B, CCL5/RANTES, CXCL9/MIG, CCL5/RANTES, BDNF and GDNF in preterm infants (*N* = 40) at three different time points (T1: 48 hours, T2: 72 hours, and T3: 3 weeks after birth). CBA was used in cytokines and chemokines ex vivo analysis, and ELISA for neurotrophic factors. Mann–Whitney test was used for comparisons between medians of patients with normal motor development versus patients of lower than expected motor development.

In urine samples, IL‐10, IL‐1*β*, CXCL9/MIG, BDNF, and GDNF values significantly increased 72 hours after birth (T2) in comparison to 48 hours after birth (T1). CCL5/RANTES and GDNF levels differed significantly between T3 and T1. BDNF levels were significantly different in all time points (Figure 3). There was no difference between time points in urine samples for IL‐12p70, TNF, IL‐6, CXCL10/IP‐10, CCL2/MCP‐1, and CXCL8/IL‐8 values.

### 3.3. Comparison of the Measurements in Plasma and Urine Samples for Inflammatory Markers and Neurotrophic Factors

To determine the relationship between the results of the measurements in plasma and urine samples, ROC curve was used. Either for plasma or urine, ROC curve results showed that no biomarker had an area under the curve (AUC) of 0.9 (Table 2). Only GDNF in plasma and urinary IL‐1*β* had AUC values of 0.8. For most of the samples, the estimated AUC was slightly higher for urine compared to plasma samples; however, confidence intervals did not show any statistical difference between them. Thus, considering the less invasive characteristics of urine collections, and the potential benefits of finding valid biomarkers for clinical use in preterm infants, we opted to use urinary concentrations of inflammatory markers and neurotrophic factors.

### 3.4. Association Between Clinical Variables And Urinary Levels of Inflammatory Markers and Neurotrophic Factors with Motor Development

TIMP was applied when participants reached at least 34 weeks of postmenstrual age in order to evaluate the motor development of the preterm. The quantification of the raw score was based on the sum of the values obtained in each of the items. Raw scores were converted into percentiles according to the standardization of development curves established by the test. In order to evaluate possible associations between motor development and other variables, TIMP results were stratified into two groups: “lower than expected” (below 5th percentile) and “typical development” (above 5th percentile). Older maternal age was associated with lower than expected TIMP scores (Table 3).

At the first time point (T0), neonates with typical motor development had higher concentrations of TNF and BDNF in the umbilical cord blood. At the other time points, plasma and urine values frequently showed similar changes. At 48 hours after birth (T1), IL‐1*β* and BDNF did not show significant differences between the two groups in both plasma and urine samples, whereas, 72 hours after birth (T2), BDNF concentrations were higher in the group with typical development. CCL5/RANTES and GDNF displayed the same behavior in both methods. However, while CCL5/RANTES levels were significantly different only in the urine samples, GDNF levels were different only in plasma samples. At three weeks postpartum (T3), GDNF showed significant difference in urine but not in plasma samples. Urinary levels of GDNF were higher in infants with lower than expected motor development (Table 4).

Regarding molecular biomarkers, the median values at three time points (T1, T2, and T3) were used. Higher urinary concentrations of BDNF and GDNF were observed in the group with lower than expected development at the time points T2 and T3, respectively. In the group with normal development, urinary IL‐1*β* levels were higher at T1, while urinary levels of CXCL8/IL‐8 were increased at T1 and T2 (Table 5).

## 4. Discussion

In this study, maternal age and urinary and plasma concentrations of inflammatory molecules and neurotrophic factors were significantly different in preterm neonates according to TIMP scores. Higher urinary levels of GDNF were found in neonates with lower than expected motor development, while IL‐1*β* and CXCL8/IL‐8 concentrations in urine were higher in those with typical motor development. There was also an association between younger maternal age and typical motor development. Sociodemographic factors including maternal age, maternal education, and maternal occupation can affect motor outcome. However, the precise relationship between these factors is not well known [33].

Pregnancy disorders that lead to preterm birth may be also associated with systematic inflammation in the newborn [34–36]. Indeed, increased concentrations of inflammatory molecules were less commonly detected in preeclampsia than other pregnancy disorders related to preterm delivery including premature placental abruption or rupture of the amniotic sac [35]. In our study, the main cause of premature delivery was preeclampsia, which was not associated with inflammatory molecules and neurotrophic factors.

The hypothesis that an inflammatory state is associated with higher incidence of brain injury is corroborated by studies showing that elevated concentrations of cytokines are associated with neuronal lesions and developmental abnormalities [15, 16, 24, 25, 37]. However, this association was not identified in other studies [38–40]. These conflicting results might be related to differences in inclusion and exclusion criteria and in the time points for the evaluation of inflammatory molecules and neurodevelopment. Specifically, an association between increased IL‐6 concentration with poorer motor development and brain abnormalities has been previously described [15, 16, 24, 41, 42]. A gene polymorphism that increases IL‐6 synthesis was associated with disabling brain injury in infants [43]. In the present study, we did not find significant differences in IL‐6 concentrations. One possible reason for the absence of changes in IL‐6 levels may be related to the fact that we have excluded neonates with confirmed or suspected sepsis, while, in other studies, babies with sepsis were included [15, 16, 24, 44, 45]. IL‐6 is a proinflammatory cytokine that increases in case of sepsis [44, 45]. The release of IL‐6 triggers an inflammatory cascade secondary to infection, with consequent developmental delay and/or brain injury [44, 46, 47]. Elevated IL‐6 is also associated with spontaneous preterm labor [34]. In this regard, it should be mentioned that all newborns were delivered by cesarean section in our study.

In contrast, higher IL‐1*β* levels were associated with better TIMP scores. Cytokines are involved in the control of many cellular interaction and cell life and are released under several stimuli [18]. IL‐1 system is associated with the recruitment of macrophages and promotes monocyte infiltration in the brain, activation of microglia and astrocytes, and production of free radicals [18, 48, 49]. However, microglial cells also play a physiological role in CNS, participating in the synaptic chopping and apoptosis that occur during development, besides actions in neurogenesis and neuronal differentiation [17, 50, 51]. Indeed, IL‐1 system has a role in cell development, differentiation, and death [18]. The balance between proinflammatory and immunomodulatory events modulates the repair/resolution processes and the occurrence of injury [17, 52]. This inflammatory response, although related to injury, is also an essential mechanism for the protection and development of CNS [50, 51]. When this response occurs and is fully resolved without brain cell death, typical abilities are maintained. On the other hand, if this response is prolonged or exacerbated, there is cell death in CNS and consequent loss of function [53].

The elevation of cytokine levels, as IL‐1*β*, is associated with the occurrence of insult, although its concentration may vary during the first hours or weeks [52]. The inflammatory response is a nonlinear system, and fluctuation in cytokines, chemokines, and neurotrophic factors levels occurred at different time points in this study. Therefore, the interaction between cytokines and CNS seems to affect motor performance [40, 54, 55]. High levels of proinflammatory cytokines correlate with white matter injury and impairment neuropsychomotor development at 2 and 3 years of age [15, 16, 42, 54, 56]. However, the relationship of these molecules with neuropsychomotor development at the first month of life was not previously evaluated.

In our results, a proinflammatory response, characterized by increased levels of IL‐1*β* in urine, was associated with typical motor development at first month of life in premature infants. This was an unexpected finding, as typically this cytokine is associated with CNS damage [35, 42, 51, 57]. However, the inflammatory response may have a physiological function in brain development and may exert protective role in the CNS [17, 50, 52, 53, 58]. This result supports the hypothesis that inflammation may have a protective effect at early stages of brain development and, until some time point, may be beneficial to the motor development. Furthermore, mutual interactions among cytokines and neurotrophic factors [7, 51, 57] may result in dynamic variations in the concentration of these molecules, in which the increase or reduction of one molecule in response to others is common [57].

GDNF is a protective factor identified as essential for the survival and neuronal differentiation, by its action on the neuroplasticity, including the modulation of neuronal survival, axon guidance, synapse formation, and functioning in the developing nervous system [56, 59–63]. GDNF concentrations can be reduced in response to increased levels of cytokines or chemokines in the brain parenchyma [56]. However, when there is an insult of the CNS, an upregulation of neurotrophic factors may occur acting as a repair mechanism [28, 64]. GDNF was an important upstream regulator of CNS, reaching high concentrations three days after an insult [64]. GDNF plays an important role in neuronal reorganization [64]. Therefore, higher levels of GDNF in the group with lower than expected motor development may be a compensatory response to a CNS insult, aiming at protecting the neurons and at inducing the formation of new synapses.

There is some evidence for a beneficial role of neuroinflammation to the CNS. Some degree of neuroinflammation is necessary for remyelination, neuroprotection, and brain development. There are several inflammatory cytokines that regulate the production of multiple neurotrophic factors by neurons and glial cells [65, 66]. This beneficial role was supported by our findings in plasma and urine samples. For instance, neonates with typical motor development had higher plasma levels of TNF and BDNF at the first time point (T0). TNF is involved in inflammation, cellular differentiation, and programmed cell death in the CNS [67]. TNF also may be beneficial during the repair stage after brain insult [66]. IL‐1*β* and TNF stimulate the release of neurotrophic factors, including BDNF and GDNF [18]. BDNF is essential for neuronal survival and differentiation, by inducing the plasticity of the CNS [68]. Indeed, complex interactions between cytokines, chemokines, and neurotrophic factors may collectively contribute to brain development and motor skill acquisition.

In order to control the influence of confounding factors, neonates presenting acute disorders were excluded from the study. The use of corticosteroids may also interfere with this process; however, as it is recommended to prevent respiratory distress syndrome [59], it was not possible to ethically avoid its administration. Also, a single course of antenatal corticosteroids was associated with reduced risk for cerebral palsy [62]. It should be mentioned that there was no difference in the results when the three neonates who were not exposed to corticosteroids were excluded from the analysis.

TIMP provides a reliable and valid measurement of the motor development that can be used for preterm neonates with 34 weeks gestational age [30–32]. This test is highly sensitive and specific. TIMP scores have significant correlation with the Bayley scale [32]. The difference between the current study and most of the published works about the association of inflammatory response and motor development may occur due to the time point in which motor development assessment was performed. Motor skill acquisition is influenced by age and cultural and contextual factors [69, 70]. For instance, at 2 years old or higher, children have been more influenced by environmental stimulus and cultural influences than at 34 weeks of gestational age. Our study reinforces the importance of assessing the newborn as early as possible in order to predict developmental abnormalities and ensure adequate interventions.

The originality of this study is the evaluation of inflammatory proteins and neurotrophic factors in spot‐urine samples as a noninvasive method of collection. In addition, samples collected in time points before motor development evaluation might predict alterations in TIMP.

## 5. Conclusion

Measurements of inflammatory biomarkers in spot‐urine samples seem to be useful in preterm neonates. This may become a noninvasive way to follow up the inflammatory profile of preterm newborns. Whether urinary levels of IL‐1*β* and of GDNF may predict motor development still needs to be confirmed.

## Figures and Tables

**Table 1:** Mother and infant characteristics.

Variables	Subjects (*n* = 40)
*Mothers*	
Preeclampsia	32 (80.0)
Other causes	8 (20.0)
Predelivery medication	
Magnesium sulfate + glucocorticoid	20 (50.0)
Glucocorticoid	17 (42.5)
None	3 (7.5)
*Infants*	
Gestational age (weeks)^#^	30 ± 1
Sex*—n* (%)	
Female	18 (45.0)
Male	22 (55.0)
Birth weight (grams)^#^	1.477 ± 428
Apgar scores^#^	
1-minute Apgar score	7 ± 1
5-minute Apgar score	9 ± 1
Respiratory distress	24 (60.0)
Exposed to antenatal glucocorticoids	37 (92.5)

^#^Values expressed as mean and standard deviation for continuous variables. Number of individuals and percentages for categorical variables.

**Table 2:** Area under the curve (AUC) values for the measurement of inflammatory markers and neurotrophic factors in plasma and urine samples.

Variables	Plasma		Urine	
	AUC values	95% confidence interval	AUC values	95% confidence interval
TNF	0.534	0.327; 0.741	0.620	0.438; 0.802
IL-12p70	0.378	0.195; 0.561	0.351	0.179; 0.523
IL-1*β*	0.561	0.373; 0.749	0.853	0.722; 0.984
CXCL10/IP-10	0.443	0.246; 0.641	0.520	0.322; 0.718
CCL2/MCP-1	0.353	0.168; 0.538	0.603	0.415; 0.791
CXCL9/ MIG	0.458	0.270; 0.645	0.559	0.364; 0.753
CCL5/RANTES	0.278	0.115; 0.442	0.568	0.388; 0.748
CXCL8/IL-8	0.556	0.356; 0.755	0.567	0.384; 0.749
BDNF	0.668	0.498; 0.839	0.418	0.230; 0.607
GDNF	0.853	0.723; 0.983	0.392	0.195; 0.589

**Table 3:** Association between clinical features and motor development results in TIMP.

Variables	Lower than expected (*N* = 22)	Typical development (*N* = 18)	*p* values
Maternal age^∗^	27.50 (24.75; 36.0)	24.00 (18.75; 28.0)	0.008^2^
Gestacional age^#^	31 ± 1	30 ± 1	0.387^1^
Birth weight^#^	1548.8 ± 479.88	1388.61 ± 347.61	0.274^1^
1-minute Apgar^∗^	7.00 (6.00; 8.00)	8.00 (5.00; 8.00)	0.967^2^
5-minute Apgar^∗^	9.00 (7.00; 9.00)	9.00 (9.00; 10.00)	0.079^2^
Sex^#^			
Female	7 (38.9)	11 (61.1)	0.064^2^
Male	15 (68.2)	7 (31.8)	

^#^Values expressed as mean and standard deviation for continuous variables. Number of individuals and percentages for categorical variables. ^∗^Median values (quartile 1; quartile 3). ^1^Student's *t*-test. ^2^Mann–Whitney test.

**Table 4:** Association between clinical features, plasma levels of inflammatory markers and neurotrophic factors, and motor development results in TIMP.

Variables	Motor development											
	T0			T1			T2			T3		
	Lower than expected (*N* = 22)	Typical development (*N* = 18)	*p*	Lower than expected (*N* = 22)	Typical development (*N* = 18)	*p*	Lower than expected (*N* = 22)	Typical development (*N* = 18)	*p*	Lower than expected (*N* = 22)	Typical development (*N* = 18)	*p*
IL-12p70	461.8550 (204.2625; 778.7525)	445.9050 (286.1100; 541.9325)	0.860	347.1500 (118.7800; 513.5800)	345.7700 (284.8100; 406.3725)	0.828	444.6450 (304.7025; 732.6200)	414.9150 (299.1000; 513.5800)	0.414	748.7100 (286.9775; 1272.8825)	456.0650 (194.6600; 633.7150)	**0.039**
TNF	1.8900 (1.8150; 2.0500)	2.0000 (1.9400; 2.3250)	**0.014**	2.1650 (2.0000; 2.3325)	2.2350 (1.9350; 2.7225)	0.957	2.3100 (2.0500; 2.5225)	2.1650 (2.0000; 2.8375)	0.614	2.1400 (2.0000; 2.2650)	2.0500 (1.8900; 2.2200)	0.169
IL-10	2.2350 (1.7275; 6.4075)	2.8700 (6.3050; 1.8650)	0.568	2.0000 (1.8900; 3.1800)	1.7400 (1.6225; 2.7975)	**0.034**	1.8900 (1.5500; 2.6950)	1.8650 (1.6400; 3.0075)	0.614	(1.7650; 4.1025)	1.9450 (1.4625; 3.5425)	0.540
IL-6	17.3700 (5.7900; 33.8100)	29.6450 (12.2050; 153.0675)	0.201	14.9600 (8.5025; 33.3300)	14.6750 (8.8650; 34.8750)	0.755	8.3900 (7.2800; 14.8775)	11.8200 (7.4925; 31.0225)	0.377	8.4250 (5.4400; 22.1100)	10.0400 (5.3400; 31.1550)	0.870
IL-1*β*	4.1900 (3.9900; 5.0825)	4.1850 (3.8175; 4.7150)	0.514	4.1900 (4.0375; 4.2800)	4.0400 (4.0000; 4.2300)	0.141	4.1600 (4.0275; 4.3800)	4.1150 (4.0000; 4.2000)	0.504	4.0900 (3.8475; 4.5925)	4.3400 (4.0675; 5.0250)	0.138
CXCL8/IL-8	49.0300 (14.0350; 172.1125)	17.1300 (12.3500; 160.4250)	0.549	23.1400 (17.0550; 53.2100)	22.5000 (14.7450; 46.9700)	0.828	15.7000 (13.8100; 46.7500)	18.2850 (15.8500; 32.4800)	0.531	13.9900 (11.6300; 146.2375)	18.8100 (12.1975; 39.1325)	0.870
CXCL10/IP-10	93.8950 (50.8550; 134.7700)	73.3200 (42.9325; 109.6675)	0.414	62.5700 (43.1425; 103.3675)	52.2150 (36.8950; 63.2425)	0.165	40.7900 (20.2825; 79.8925)	52.2800 (36.8600; 57.2125)	0.369	59.9800 (38.9750; 123.0900)	46.0900 (21.9350; 109.9275)	0.765
CCL2/MCP-1	141.3000 (48.9550; 348.1200)	138.5350 (40.8100; 265.5500)	0.849	173.6600 (84.9775; 269.8575)	92.0600 (32.5650; 173.9875)	0.103	169.0100 (63.1475; 257.2900)	80.6350 (22.8975; 131.7525)	0.075	229.8450 (50.4000; 516.4825)	156.3050 (56.1325; 409.2650)	0.584
CXCL9/ MIG	12.5300 (9.5500; 17.4075)	10.9300 (7.4425; 21.9300)	0.605	12.1650 (9.0025; 17.7350)	9.5500 (6.5550; 15.1825)	0.301	11.0600 (7.1300; 18.1100)	12.1000 (9.2250; 14.6475)	0.849	11.4100 (6.6400; 68.6350)	13.7250 (5.6100; 18.1100)	0.786
CCL5/RANTES	4189.0300 (3490.1500; 4912.6700)	3108.0400 (2565.0250; 4522.1000)	0.108	3280.4350 (1714.4400; 5047.0700)	3234.7900 (1013.3375; 3994.4200)	0.341	4140.9550 (2707.1900; 4890.3275)	1246.8250 (819.4700; 3959.1275)	**0.002**	4484.2700 (4254.0700; 4923.7700)	4429.8650 (3055.6900; 4654.5300)	0.173
BDNF	58,67050 (41,87350; 127,22950)	136,90750 (52,52600; 291,69825)	**0.044**	128,28300 (69,72725; 311,45300)	175,39450 (65,41075; 252,36550)	0.946	97,98350 (48,77400; 198,88925)	211,71850 (117,52025; 400,48775)	**0.036**	88,00000 (41,60925; 341,58475)	141,65450 (70,52900; 303,92975)	0.145
GDNF	233,97500 (81,72825; 257,72450)	233,97300 (199,83950; 300,28300)	0.216	123,24200 (24,76400; 338,97525)	239,41100 (146,92575; 297,91550)	0.178	130,52900 (101,50500; 166,84500)	217,26600 (151,14075; 271,96400)	**0.000**	115,9175 (26,9508; 520,3155)	130,4910 (30,9778; 526,7730)	0.634

Median values (quartile 1; quartile 3). Mann–Whitney test. T0: umbilical cord blood; T1: 48 hours; T2: 72 hours; T3: 3 weeks after birth.

Bold characters of *p* values refer to statistically significant differences.

**Table 5:** Association between clinical features, urinary levels of inflammatory markers and neurotrophic factors and motor development results in TIMP.

Variables	Motor development								
	T1			T2			T3		
	Lower than expected (*N* = 22)	Typical development (*N* = 18)	*p*	Lower than expected (*N* = 22)	Typical development (*N* = 18)	*p*	Lower than expected (*N* = 22)	Typical development (*N* = 18)	*p*
IL-12p70	2.590 (2.400; 2.750)	2.460 (2.393; 2.568)	0.119	2.520 (2.393; 2.668)	2.475 (2.445; 2.575)	0.757	2.490 (2.370; 2.600)	2.430 (2.355; 2.475)	0.476
TNF	1.765 (1.665; 1.955)	1.690 (1.590; 1.803)	0.180	1.640 (1.550; 1.840)	1.765 (1.580; 1.903)	0.251	1.590 (1.590; 1.715)	1.665 (1.550; 1.840)	0.443
IL-10	0.965 (0.880; 1.130)	1.040 (0.930; 1.100)	0.381	1.090 (0.918; 1.328)	1.040 (0.980; 1.240)	0.638	1.000 (0.980; 1.455)	0.990 (0.930; 1.090)	0.299
IL-6	4.140 (3.860; 5.578)	4.235 (3.983; 6.910)	0.667	4.835 (3.868; 7.105)	4.105 (3.770; 4.780)	0.262	4.520 (3.780; 11.495)	4.050 (3.505; 8.060)	0.199
IL-1*β*	4.380 (4.155; 5.800)	5.790 (5.138; 7.925)	**0.012** ^∗^	5.440 (4.520; 9.090)	7.095 (6.015; 10.045)	0.079	5.190 (4.620; 7.980)	5.985 (4.855; 8.730)	0.527
CXCL8/IL-8	8.415 (6.370; 32.500)	25.710 (11.213; 60.118)	**0.032** ^∗^	8.910 (7.743; 29.780)	33.095 (15.918; 55.230)	**0.045** ^∗^	11.360 (7.820; 40.405)	17.570 (7.628; 53.986)	0.925
CXCL10/IP-10	2.110 (0.680; 5.320)	4.190 (0.680; 8.980)	0.925	2.310 (0.980; 13.230)	5.26 (2.783; 10.915)	0.396	1.945 (0.680; 17.430)	4.475 (1.875; 11.950)	0.427
CCL2/MCP-1	24.890 (23.580; 103.805)	40.555 (10.545; 104.098)	0.798	54.230 (23.333; 123.120)	66.320 (33.910; 96.363)	0.510	46.675 (20.560; 154.975)	45.380 (20.885; 67.118)	0.819
CXCL9/ MIG	1.035 (0.733; 1.955)	1.140 (0.780; 1.920)	0.581	1.670 (1.040; 2.408)	1.485 (1.078; 2.130)	0.840	1.220 (0.660; 5.730)	1.250 (0.785; 8.805)	0.677
CCL5/RANTES	5.920 (4.535; 7.103)	5.590 (4.168; 6.350)	0.600	5.650 (3.525; 7.628)	4.600 (3.700; 7.660)	0.989	3.635 (3.030; 4.355)	4.74 (4.145; 6.100)	**0.020** ^∗^
BDNF	11.271 (0.0; 36.345)	14.081 (3.017; 17.922)	0.600	54.083 (31.736; 61.840)	24.830 (0.0; 53.813)	**0.039** ^∗^	20.226 (13.312; 27.131)	27.131 (16.387; 36.350)	0.112
GDNF	26.023 (17.923; 62.471)	26.927 (20.486; 56.181)	0.798	58.473 (28.887; 91.789)	117.092 (23.158; 251.53)	0.254	102.304 (52.843; 430.570)	38.340 (18.75; 64.400)	**0.005** ^∗∗^

Median values (quartile 1; quartile 3). Mann–Whitney test. T1: 48 hours; T2: 72 hours; T3: 3 weeks after birth.

^*^ *p* < 0.05 and ^**^p < 0.01.

## References

[1] Kemp M. W. (2014). Preterm birth, intrauterine infection, and fetal inflammation. *Frontiers in Immunology*.

[2] van Vliet E. O., de Kieviet J. F., Oosterlaan J., van Elburg R. M. (2013). Perinatal infections and neurodevelopmental outcome in very preterm and very low‐birth‐weight infants: a meta‐analysis. *JAMA Pediatrics*.

[3] Logitharajah P., Rutherford M. A., Cowan F. M. (2009). Hypoxic‐ischemic encephalopathy in preterm infants: antecedent factors, brain imaging, and outcome. *Pediatric Research*.

[4] Guo R., Hou W., Dong Y., Yu Z., Stites J., Weiner C. P. (2010). Brain injury caused by chronic fetal hypoxemia is mediate by inflammatory cascade activation. *Reproductive Sciences*.

[5] Selip D. B., Jantzie L. L., Chang M. (2012). Regional differences in susceptibility to hypoxic‐ischemic injury in the preterm brain: exploring the spectrum from with matter loss to selective grey matter injury in a rat model. *Neurology Research International*.

[6] Degos V., Favrais G., Kaindl A. M. (2010). Inflammation process in perinatal brain damage. *Journal of Neural Transmission*.

[7] Malaeb S., Damann O. (2009). Fetal inflammatory response and brain injury in the preterm newborn. *Journal of Child Neurology*.

[8] Berguer I., Preleg O., Ofek-Shlomai N. (2012). Inflammation and early brain injury in term and preterm infants. *The Israel Medical Association Journal*.

[9] Khwaja O., Volp J. J. (2008). Pathogenesis of cerebral white matter injury of prematurity. *Archives of Disease in Childhood. Fetal and Neonatal Edition*.

[10] Deng W. (2010). Neurobiology of injury to the developing brain. *Nature Reviews. Neurology*.

[11] Brochu M. E., Girard S., Lavoie K., Sébire G. (2011). Developmental regulation of the neuroinflammatory responses to LPS and/or hypoxia-ischemia between preterm and term neonates: an experimental study. *Journal of Neuroinflammation*.

[12] Leitner K., Al Shammary M., McLane M., Johnston M. V., Elovitz M. A., Burd I. (2014). IL‐1 receptor blockade prevents fetal cortical brain injury but not preterm birth in a mouse model of inflammation‐induced preterm birth and perinatal brain injury. *American Journal of Reproductive Immunology*.

[13] Adén U., Favrais G., Plaisant F. (2010). Systemic inflammation sensitizes the neonatal brain to excitotoxicity through a pro‐/anti‐inflammatory imbalance: key role of TNF‐alpha pathway and protection by etanercept. *Brain, Behavior, and Immunity*.

[14] Girard S., Sébirre G., Kadhim H. (2010). Proinflammatory orientation of the interleukin 1 system and downstream induction of matrix metalloproteinase 9 in the pathophysiology of human perinatal white matter damage. *Journal of Neuropathology and Experimental Neurology*.

[15] O’Shea T. M., Shah B., Allred E. N. (2013). Inflammation‐initiating illnesses, inflammation‐related proteins, and cognitive impairment in extremely preterm infants. *Brain, Behavior, and Immunity*.

[16] O’Shea T. M., Allred E. N., Kuban K. C. (2012). Elevated concentrations of inflammation‐related proteins in postnatal blood predict severe developmental delay at two years in extremely preterm infants. *The Journal of Pediatrics*.

[17] Hagberg H., Gressens P., Mallard C. (2012). Inflammation during fetal and neonatal life: implications for neurologic and neuropsychiatric disease in children and adults. *Annals of Neurology*.

[18] Girard S., Kadhim H., Roy M. (2009). Role of perinatal inflammation in cerebral palsy. *Pediatric Neurology*.

[19] Jin C., Londono I., Mallard C., Lodygensky G. A. (2015). New means to assess neonatal inflammatory brain injury. *Journal of Neuroinflammation*.

[20] de Kieviet J. F., Zoetebier L., Van Elburg R. M., Vermeulen R. J., Oosterlaan J. (2012). Brain development of very preterm and very low‐birth weight children in childhood and adolescence: a meta‐analysis. *Developmental Medicine and Child Neurology*.

[21] Linsell L., Malouf R., Morris J., Kurinczuk J. J., Marlow N. (2015). Prognostic factors for poor cognitive development in children born very preterm or with very low birth weight: a systematic review. *JAMA Pediatrics*.

[22] Ferrari F., Gallo C., Pugliese M. (2012). Preterm birth and developmental problems in the preschool age. Part I: minor motor problems. *The Journal of Maternal‐Fetal & Neonatal Medicine*.

[23] Alvarez‐Diaz A., Hilario E., Goni de Cerio F., Valls‐i‐Soler A., Alvarez‐Diaz F. J. (2007). Hypoxic‐ischemic injury in the immature brain: key vascular and cellular players. *Neonatology*.

[24] Duggan P. J., Maalouf E. F., Watts T. L. (2001). Intrauterine T‐cell activation and increased proinflammatory cytokine concentrations in preterm infants with cerebral lesions. *Lancet*.

[25] Heep A., Behrendt D., Nitsch P., Fimmers R., Bartmann P., Dembinski J. (2003). Increased serum levels of the interleukin 6 are associated with severe intraventricular haemorrhage in extremely premature infants. *Archives of Disease in Childhood. Fetal and Neonatal Edition*.

[26] Risso F. M., Sannia A., Gavilanes D. A. (2012). Biomarkers of brain damage in preterm infants. *The Journal of Maternal‐Fetal & Neonatal Medicine*.

[27] Chang E. (2015). Preterm birth and the role of neuroprotection. *BMJ*.

[28] Fantacci C., Capozzi D., Ferrara P., Chiaretti A. (2013). Neuroprotective role of nerve growth factor in hypoxic‐ischemic brain injury. *Brain Sciences*.

[29] Campbell S. K., Kolobe T. H., Osten E. T., Lenke M., Girolami G. L. (1995). Construct validity of the test of infant motor performance. *Physical Therapy*.

[30] Barbosa V. M., Campbell S. K., Berbaum M. (2007). Discriminating infants from different developmental outcome groups using the test of infant motor performance (TIMP) item responses. *Pediatric Physical Therapy*.

[31] Snider L., Majnemer A., Mazer B., Campbell S., Bos A. F. (2009). Prediction of motor and functional outcomes in infants born preterm assed at term. *Pediatric Physical Therapy*.

[32] Kim S. A., Lee Y. J., Lee Y. G. (2011). Predictive value of test of infant motor performance for infants based on correlation between TIMP and Bayley scales of infant development. *Annals of Rehabilitation Medicine*.

[33] Patra K., Greene M. M., Patel A. L., Meier P. (2016). Maternal education level predicts cognitive level, and motor outcome in preterm infants in the second year of life. *American Journal of Perinatology*.

[34] Ferguson K. K., TF M. E., Chen Y. H., Mukherjee B., Meeker J. D. (2014). Longitudinal profiling of inflammatory cytokines and C‐reactive protein during uncomplicated and preterm pregnancy. *American Journal of Reproductive Immunology*.

[35] Kahyaoğlu S., Timur H., Eren R., Kahyaoğlu İ., Eyi E. G., Engin‐Üstün Y. (2014). Can maternal serum C‐reactive protein levels predict successful labour induction with intravenous oxytocin in term pregnancies complicated with premature rupture of the membranes? A cross‐sectional study. *Journal of the Turkish German Gynecological Association*.

[36] McElrath T. F., Fichorova R. N., Allred E. N. (2011). Blood protein profiles of infants born before 28 weeks differ by pregnancy complication. *American Journal of Obstetrics and Gynecology*.

[37] Lodha A., Asztalos E., Moore A. M. (2010). Cytokine levels in neonatal necrotizing enterocolitis and long-term growth and neurodevelopment. *Acta Paediatrica*.

[38] Silveira R. C., Procianoy R. S. (2011). High plasma cytokine levels, with matter injury and neurodevelopment of high risk preterm infants: assessment at two years. *Early Human Development*.

[39] Varner M. W., Marshall N. E., Rouse D. J. (2015). The association of cord serum cytokines with neurodevelopmental outcomes. *American Journal of Perinatology*.

[40] Carlo W. A., SA M. D., Tyson J. E. (2011). Cytokines and neurodevelopmental outcomes in extremely low birth weight infants. *The Journal of Pediatrics*.

[41] Viscardi R. M., Muhumuza C. K., Rodriguez A. (2004). Inflammatory markers in intrauterine and fetal blood and cerebrospinal fluid compartments are associated with adverse pulmonary and neurologic outcomes in preterm infants. *Pediatric Research*.

[42] Korzeniewski S. J., Soto-Rivera C. L., Fichorova R. N. (2014). Are preterm newborns who have relative hyperthyrotropinemia at increased risk of brain damage?. *Journal of Pediatric Endocrinology & Metabolism*.

[43] Harding D. R., Dhamrait S., Whitelaw A., Humphries S. E., Marlow N., Montgomery H. E. (2004). Does interleukin‐6 genotype influence cerebral injury or developmental progress after preterm birth?. *Pediatrics*.

[44] Chiesa C., Pacifico L., Natale F., Hofer N., Osborn J. F., Resch B. (2015). Fetal and early neonatal interlukin‐6 response. *Cytoline*.

[45] Shahkar L., Keshtkar A., Mirfazeli A., Ahani A., Roshandel G. (2011). The role of IL‐6 for predicting neonatal sepsis: a systematic review and meta-analysis. *Iranian Journal of Pediatrics*.

[46] Yoon B. H., Park C. W., Chaiworaponsa T. (2003). Intrauterine infection and the development of cerebral palsy. *Bjog*.

[47] Adams‐Chapman I., Stoll B. J. (2006). Neonatal infection and long-term neurodevelopment outcome in the preterm infant. *Current Opinion in Infectious Diseases*.

[48] Stojkovska I., Wagner B. M., Morrison B. E. (2015). Parkinson’s disease and enhanced inflammatory response. *Experimental Biology and Medicine*.

[49] Chanaday N. L., Roth G. A. (2016). Microglia and astrocyte activation in the frontal cortex of rats with experimental autoimmune encephalomyelitis. *Neuroscience*.

[50] Mallard C., Davidson J. O., Tan S. (2014). Astrocytes and microglia in acute cerebral injury underlying cerebral palsy associate with preterm birth. *Pediatric Research*.

[51] Ranchhod S. M., Gunn K. C., Fowke T. M. (2015). Potential protective strategies for perinatal infection and inflammation. *International Journal of Developmental Neuroscience*.

[52] Savard A., Lavoie K., Brochu M. E. (2013). Involvement of neuronal IL‐1*β* in acquired brain lesions in a rat model of neonatal encephalopathy. *Journal of Neuroinflammation*.

[53] McCusker R. H., Kelley K. W. (2013). Immune-neural connections: how the immune system’s response to infections agents influences behavior. *The Journal of Experimental Biology*.

[54] Leviton A., Fichorova R. N., O’Shea T. M. (2013). Two-hit model of brain damage in the very preterm newborn: small for gestational age and postnatal systemic inflammation. *Pediatric Research*.

[55] Pajenda G., Hercher D., Márton G. (2014). Spatiotemporally limited BDNG and GDNF over expression rescues motoneurons destined to die and induces elongative axon growth. *Experimental Neurology*.

[56] Kinjo T., Ohga S., Ochiai M. (2011). Serum chemokine levels and developmental outcome in preterm infants. *Early Human Development*.

[57] Kuban K. C., O’Shea T. M., Allred E. N. (2015). The breadth and type of systemic inflammation and the risk of adverse neurological outcome in extremely low gestation newborns. *Pediatric Neurology*.

[58] Chhor V., Le Charpentier T., Lebon S. (2013). Characterization of phenotype markers and neuronotoxic potential of polarized primary microglia in vitro. *Brain, Behavior, and Immunity*.

[59] Pius-Sadowska E., Kawa M. P., Kłos P. (2016). Alteration of neurotrophic factors and their reception expression in mouse brain response to whole-brain irradiation. *Radiation Research*.

[60] Spulber S., Rantamäki T., Nikkilä O. (2010). Effects of maternal smoking and exposure to Methylmercury on brain‐derived neurotrophic factor concentrations in umbilical cord serum. *Toxicological Sciences*.

[61] Avdoshina V., Becker J., Campbell L. A. (2011). Neurotrophins modulate the expression of chemokine receptors in brain. *Journal of Neurovirology*.

[62] Roberts D., Dalziel S. (2006). Antenatal corticosteroids for accelerating fetal lung maturation for women at risk of preterm birth. *The Cochrane Database of Systematic Reviews*.

[63] Sotiriadis A., Tsiami A., Papatheodorou S., Baschat A. A., Sarafidis K., Makrydimas G. (2015). Neurodevelopmental outcome after a single course of antenatal steroids in children born preterm: a systematic review and meta-analysis. *Obstetrics and Gynecology*.

[64] Kim J. H., Kim S. H., Cho S. R. (2016). The modulation of neurotrophin and epigenetic regulators: implication of astrocyte proliferation and neuronal cell apoptosis after spinal cord injury. *Annals of Rehabilitation Medicine*.

[65] Rosa L., Scaini G., Furlanetto C. B. (2016). Administration of branched‐chain amino acids alters the balance between pro-inflammatory and anti-inflammatory cytokines. *International Journal of Developmental Neuroscience*.

[66] Wee Yong V. (2010). Inflammation in neurological disorders: a help or a hindrance?. *The Neuroscientist*.

[67] Babri S., Doosti M. H., Salari A. A. (2014). Tumor necrosis factor‐alpha during neonatal brain development affects anxiety‐ and depression‐related behaviors in adult male and female mice. *Behavioural Brain Research*.

[68] Uguz F., Sonmez E. O., Sahingoz M. (2013). Maternal generalized anxiety disorder during pregnancy and fetal brain development: a comparative study on cord blood brain‐derived neurotrophic factor levels. *Journal of Psychosomatic Research*.

[69] Santos V. M., Formiga C. K. M. R., Mello P. R. B., Leone C. R. (2017). Late preterm infants’ motor development until term age. *Clinicas (São Paulo)*.

[70] Nuysink J., van Haastert I. C., Eijsermans M. J. (2013). Prediction of gross motor development and independent walking in infants born very preterm using the test of infant motor performance and the Alberta infant motor scale. *Early Human Development*.

